# Minimum Dietary Diversity Among Children Aged 6–59 Months in East Africa Countries: A Multilevel Analysis

**DOI:** 10.3389/ijph.2023.1605807

**Published:** 2023-06-01

**Authors:** Temam Beshir Raru, Bedasa Taye Merga, Gutema Mulatu, Alemayehu Deressa, Abdi Birhanu, Belay Negash, Mulugeta Gamachu, Lemma Demissie Regassa, Galana Mamo Ayana, Kedir Teji Roba

**Affiliations:** ^1^ School of Public Health, College of Health and Medical Sciences, Haramaya University, Harar, Ethiopia; ^2^ Department of Environmental Health, College of Health and Medical Sciences, Haramaya University, Harar, Ethiopia; ^3^ School of Medicine, College of Health and Medical Sciences, Haramaya University, Harar, Ethiopia; ^4^ Departments of Public Health, Rift Valley University, Harar, Ethiopia; ^5^ School of Nursing and Midwifery, College of Health and Medical Sciences, Haramaya University, Harar, Ethiopia

**Keywords:** multilevel analysis, dietary diversity, East Africa, DHS, 6–59 months

## Abstract

**Objective:** To find out the determinants of minimum dietary diversity (MDD) among under-five children in East Africa based on the 2017 revised indicator.

**Methods:** Secondary data from the demographic and health survey (DHS) of eight countries in East Africa were combined. A total of 27,223 weighted samples of children aged 6–59 months were included. Multi-level logistic regression analysis was employed to identify the determinants of dietary diversity.

**Results:** The magnitude of adequate MDD in East Africa was found to be 10.47% with 95% CI (10.12–10.84) with the lowest and highest magnitude in Ethiopia and Rwanda respectively. Having a mother in the age group of 35–49, having a mother with higher educational attainment, and having a post-natal check-up within 2 months were significant factors in determining adequate MDD.

**Conclusion:** The magnitude of adequate MDD intake among children aged 6–59 months in East Africa is relatively low. Therefore, strengthening interventions focused on improving the economic status of households, the educational status of mothers, and diversified food consumption of children aged 6–59 months should get priority to improve the recommended feeding practice of children.

## Introduction

Dietary diversity is a quantitative number of food items or groups used as a method of determining the variety and nutrient adequacy of diets for an individual [1]. The world health organization (WHO) recommends that children should consume a diverse range of nutritionally balanced, suitable, and healthy foods to meet their nutritional requirements [2]. However, globally, less than a quarter of children aged 6–23 months satisfy the recommended standards for dietary diversity, and only a small portion of them receive a diet that is nutritionally sufficient [3].

Of the total 10.9 million deaths among children under the age of 5, malnutrition is directly or indirectly accountable for 60% of these fatalities. Each year, over 3.4 million children under the age of 5 die as a result of inadequate feeding practices. Among these deaths, approximately two-thirds are linked to inappropriate feeding practices that occur within the first 2 years of life [4, 5]. Less than one-quarter of children are reported to be malnourished in many nations, especially during the important first 1,000 days of life [6, 7]. The prevalence of adequate minimum dietary diversity was 46.5% in Nepal [8], 23%–63.85% in India [9, 10], 53.3% in Indonesia [11], and 71% in Sri Lanka [12].

In sub-Saharan African countries, the overall prevalence of minimum dietary diversity was 25.1%, with the highest and lowest in South Africa (43.9%) and Burkina Faso (5.6%), respectively [13] and in Ethiopia, it was 23.25% among children age 6–23 months [14].

The United Nations Sustainable Development Goals (SDGs) aim to enhance the health and wellbeing of all children by eliminating preventable deaths among newborns and children under the age of five by the year 2030. In order to achieve this objective, it is crucial for the SDGs to place a strong emphasis on nutrition priorities. Improvements in child nutrition play a significant role in attaining these sustainable development goals [15]. Dietary diversity is a good predictor of dietary quality and micronutrient density. Children residing in low and middle-income countries are known to experience micronutrient deficiencies, primarily caused by poor diet quality [16, 17]. Inadequate dietary diversity is a significant issue among impoverished populations in the developing world, particularly in Africa [18].

Attaining the minimum dietary diversity (MDD) for children under the age of five in sub-Saharan Africa (SSA) presents a major challenge due to the prevalence of poverty. Many parents in the region face low-income conditions, which hinder their ability to afford and provide appropriate complementary feeding practices for their infants, as well as meet the minimum requirements for dietary diversity among young children [19, 20]. For children who are not breastfed, it is recommended to provide them with meals four or five times a day, along with one to two snacks as desired. The frequency of meals serves as an indicator of energy intake from sources other than breast milk. Thus, when assessing feeding frequency for non-breastfed children, both milk feeds and solid or semi-solid feeds are taken into account [21, 22].

Shreds of evidence revealed maternal healthcare utilization status, sociodemographic factors like maternal education, residence, mother’s occupation, economic factors, and media exposure of the mothers were identified as determinants of dietary diversity [23–28].

So far, many small-scale studies have been conducted on child dietary diversity among children under five, but based on a cut-off of four out of seven food groups. The indicator was revised in 2017 to add breast milk as a separate food group, thereby increasing the total number of food groups to eight and increasing the cut-off to five groups. The indicator was revised because the previous indicator included infant formula but not breast milk. Therefore, this study is intended to find out the determinants of dietary diversity among children under five in East Africa based on the revised indicator.

## Methods

### Data Source, Tool, and Sampling Procedure

The data for this research was taken from the Measure Demographic and Health Survey (DHS) program, which can be accessed at www.measuredhs.com. The DHS program covers over 90 countries with low- and middle-income economies, allowing for a comprehensive collection of data. The data obtained from the program is designed to be comparable across different countries, enabling meaningful cross-country analysis and comparison. This study strictly adhered to the relevant statistical guidelines established by the DHS program. The program ensured uniformity by employing the same manual, data collection instrument, variable names, variable codes, and sampling process across all participating countries. By following these standardized protocols, the study maintains consistency and upholds the integrity of the data [29]. Data from the DHS were combined from 2015 to 2020 for eight countries in East Africa. During the designated period, the most recent DHS of Country-specific datasets were extracted. The 8 East Africa Countries from which data were extracted with their corresponding DHS year are Burundi (2017), Ethiopia (2016), Malawi (2016), Rwanda (2020), Tanzania (2016), Uganda (2016), Zambia (2018), and Zimbabwe (2015). The DHS program ensures the collection of comparable data across countries worldwide through the adoption of standardized methods. This includes the use of uniform questionnaires, manuals, and field procedures. The surveys conducted by DHS are nationally representative household surveys that involve face-to-face interviews with women aged 15–49 years. These surveys provide comprehensive information on various indicators related to population, health, and nutrition, making them valuable for monitoring and evaluating impacts in these areas. The surveys apply a stratified, multi-stage, random sampling design. Information was obtained from eligible women aged 15–49 years in each country. Survey methodology and sampling methods used to gather the data have been described in detail elsewhere [30].

### Population and Sample Size

The source population for this study was all children aged 6–59 months in East Africa, whereas all children aged 6–59 months in East Africa who were in the selected countries were the study population. Specific children who have no record of the outcome variable were excluded.

The data sets were extracted from kids record (KR) files, which contain information about women and children, for this specific research, and extracting important variables related to adequate dietary diversity were performed from the data set. Accordingly, a total weighted sample of 27,223 who fulfilled the inclusion criteria was included in this study. This includes Burundi (3,592), Ethiopia (5,165), Malawi (2,388), Rwanda (1,895), Tanzania (4,938), Uganda (2,401), Zambia (4,370), and Zimbabwe (2,473).

### Measurement

The outcome variable of this study was Minimum Dietary Diversity (MDD). MDD was measured as the percentage of children aged 6–59 months who have consumed foods and beverages from at least five out of eight defined food groups within the previous day. The outcome variable is binary, and it is coded as 1 if the children consumed at least five food categories in the last 24 h before an interview, were considered to have met the MDD requirements, and their MDD score was categorized as having adequate MDD. The variable is coded as 0 if the children consumed less than five food categories in the last 24 h before an interview, were considered to have not met the MDD requirements, and their MDD score was categorized as having inadequate MDD.

Based on different kinds of literature we have included two types of independent variables that are individual-level and community-level variables. The individual-level variables are the age of a child, sex of the child, age of the mother, mother’s age at 1st birth, marital status, educational status of the mother, occupational status of the mother, parity, family size, wealth index, number of ANC visits, media exposure, birth order, place of delivery, and child meal frequency. The community-level variables are residence, community-level education, and country.

**Media Exposure:** was determined based on the respondents’ exposure to newspapers/magazines, radio, and television. If the respondents reported using at least one of these media sources, they were considered to have media exposure.

**Community-level Education:** was constructed by combining individual-level variables to capture the impact of the neighborhood or community on minimum dietary diversity. Individual-level values were aggregated at the cluster level to create the community-level variable. The aggregated variables were then categorized as either “high” or “low” based on the distribution of proportion values calculated for each cluster or community.

### Data Management and Analysis

To ensure the survey’s representativeness and account for the sampling design when calculating standard errors and providing valid statistical estimates, the data were weighted using various factors. These factors include sampling weights, primary sampling units, and strata. Before any statistical analysis was conducted, these weights were applied to the data. We have used the sampling weight from DHS individual sample weights which are generated by dividing (v005) by 1,000,000 before using it to approximate the number of cases. STATA version 17 was used for analysis.

### Multi-Level Analysis

Given the hierarchical nature of DHS data, there is a possibility of a cluster effect, which can violate the assumption of independent observations and equal variance across clusters. To account for this, and considering the binary nature of the outcome variable, a two-level mixed-effects logistic regression analysis was conducted. This approach allows for the consideration of between-cluster variability and helps to obtain reliable standard errors and unbiased estimates. By incorporating random effects at the cluster level, the analysis properly addresses the potential clustering effect, providing a more accurate assessment of the relationship between variables and the outcome of interest [31, 32]. In the analysis of determinants for Minimum Dietary Diversity (MDD), the individual and community-level variables were initially examined independently using a bivariable multilevel logistic regression model. In this step, variables were assessed for their association with MDD, and those that demonstrated statistical significance at a *p*-value of 0.2 in the bivariable analysis were selected for the individual and community level model adjustments. Finally, a multivariable multilevel mixed-effects analysis was conducted to identify the significant determinants of Minimum Dietary Diversity (MDD). In this analysis, variables that exhibited a *p*-value of ≤0.05 were considered statistically significant determinants of MDD.

### Model Building

Four models were fitted in the analysis. The first model, referred to as the null model, did not include any exposure variables. Its purpose was to assess the variation at the community level and provide evidence to evaluate random effects at the community level. The second model was the adjustment of the multiple variable model for individual variables and the third model was adjusted to consider factors at the community level. Whereas, in the fourth model, potential candidate variables from individual and community variables were adjusted to the outcome variable. The model with smaller AIC and BIC was considered as a parsimonious model.

### Parameter Estimation Method

Fixed effects were employed to estimate the association between the probability of Minimum Dietary Diversity (MDD) and explanatory variables at both the community and individual levels. The results of this estimation were expressed as odds ratios along with their corresponding 95% confidence intervals.

In assessing the measures of variation or random effects in the analysis, several indicators were utilized, including the intra-cluster correlation coefficient (ICC), the proportional change in variance (PCV), and the median odds ratio (MOR).

The intra-cluster correlation coefficient (ICC) measures the proportion of the total variation in Minimum Dietary Diversity (MDD) outcomes that is attributable to differences between communities. A higher ICC value indicates greater clustering or similarity of MDD outcomes within communities.

The proportional change in variance (PCV) quantifies the percentage of variation in MDD outcomes explained by the inclusion of explanatory variables in the model. It provides insights into the extent to which the individual and community-level variables account for the observed variation in MDD.

The median odds ratio (MOR) is a measure that assesses the extent of between-community variation by comparing the odds of MDD for two randomly chosen individuals from different communities. It provides an understanding of the potential influence of unmeasured community-level factors on MDD.

The purpose of the Median Odds Ratio (MOR) is to translate the area level variance in the widely used odds ratio (OR) scale that has a consistent and intuitive interpretation.

It is computed by; $MOR=\exp\left(\sqrt{2*V_{A}}*0.6745\right)$

$$MOR\approx \exp\left(0.95\sqrt{V_{A}}\right)$$

Where; VA is the area level variance, and 0.6745 is the 75th centile of the cumulative distribution function of the normal distribution with mean 0 and variance 1. See elsewhere for a more detailed explanation. Whereas the PCV is calculated as:

PCV=VA−VBVA*100 (1)

Where; where $V_{A}$ = variance of the initial model, and $V_{B}$ = variance of the model with more terms.

By using these measures of variation, the study aimed to evaluate the clustering effect and assess the impact of individual and community-level variables on the observed variation in MDD outcomes.

## Results

### Socio-Demographic and Economic Characteristics

A total of 27,223 children aged 6–59 months were included in this study. Most of the children 9,650 (35.45%) were between the age of 6–15 months, and the mean age of the child with a standard deviation is 25.83 ± 16.12 months. Exactly, half 50.0% of the children’s mothers had a primary education. The majority of the children 22,130 (81.29%) were from rural areas. The largest number of children 5,165 (18.97%) were from Ethiopia, while the smallest number of children 1,895 (6.96%) were from Rwanda (Table 1).

**TABLE 1:** Socio-demographic and economic characteristics of under-five children in East Africa countries from 2015 to 2020.

Variables	Weighted frequency	Percentage (%)
Age of Child (Months)		
6–23	16,578	61.09
24–59	10,559	38.91
Sex of Child		
Female	13,602	49.97
Male	13,620	50.03
Mothers Age (Years)		
15–24	8,215	30.18
25–34	13,290	48.82
35–49	5,717	21.00
Marital Status		
Not Married	1,180	4.33
Ever Married	26,042	95.67
Mothers Education		
No Education	7,430	27.30
Primary	13,611	50.00
Secondary	5,467	20.08
Higher	714	2.62
Mothers occupation		
No Occupation	8,883	32.63
Had Occupation	18,339	67.37
Sex of HH Head		
Female	21,899	80.45
Male	5,323	19.55
Wealth Index		
Poor	13,110	48.16
Middle	5,359	19.69
Rich	8,753	32.15
Media Exposure		
No	11,414	41.93
Yes	15,808	58.07
Partner Education (24,209)		
No Education	5,394	22.28
Primary	12,042	49.74
Secondary	5,612	23.18
Higher	1,161	4.79
Number of HH Members		
≤5	12,135	44.58
6 and Above	15,087	55.42
Number of under 5 children		
≤2	20,741	76.19
3 and Above	6,481	23.81
Age at 1st birth		
<20	16,324	59.00
20–30	11,079	40.05
31–49	263	0.95
Community Level Education		
Lower	6,379	23.43
Higher	20,843	76.57
Residence		
Rural	22,130	81.29
Urban	5,092	18.71
Country		
Burundi	3,592	13.20
Ethiopia	5,165	18.97
Malawi	2,388	8.77
Rwanda	1,895	6.96
Tanzania	4,938	18.14
Uganda	2,401	8.82
Zambia	4,370	16.05
Zimbabwe	2,473	9.08

### Service-Related Characteristics

Nearly half (45.43%) of the mothers did not have any ANC visits at all. The majority of the mothers (18,563, 68.19%) delivered at a health facility. Only 5,885 (36.35%) mothers of children aged 6–59 months had Post-natal checks within 2 months. Most of the children (5,990, 36.89%) had their meal twice per day (Table 2).

**TABLE 2:** Service related characteristics of children under five in East Africa countries from 2015 to 2020.

Variables	Weighted frequency	Percentage (%)
Number of ANC Visit		
No Visit	12,366	45.43
1–3 Visit	6,268	23.02
4 and above visit	8,588	31.55
Child Twin		
No	26,585	97.66
Yes	637	2.34
Birth Order		
First Order	6,350	23.33
Two to Fourth order	13,104	48.14
Fifth and above order	7,768	28.54
Place of Delivery		
Home	8,659	31.81
Health Institution	18,563	68.19
Post-natal check with in 2 months (*n* = 16,189)		
No	10,304	63.65
Yes	5,885	36.35
Meal Frequency (*n* = 16,236)		
One Time	3,792	23.35
Two Time	5,990	36.89
Three Time	4,419	27.22
Four and above	2,035	12.54

### The Proportion of Each of the Eight Food Groups

**FIGURE 1:**
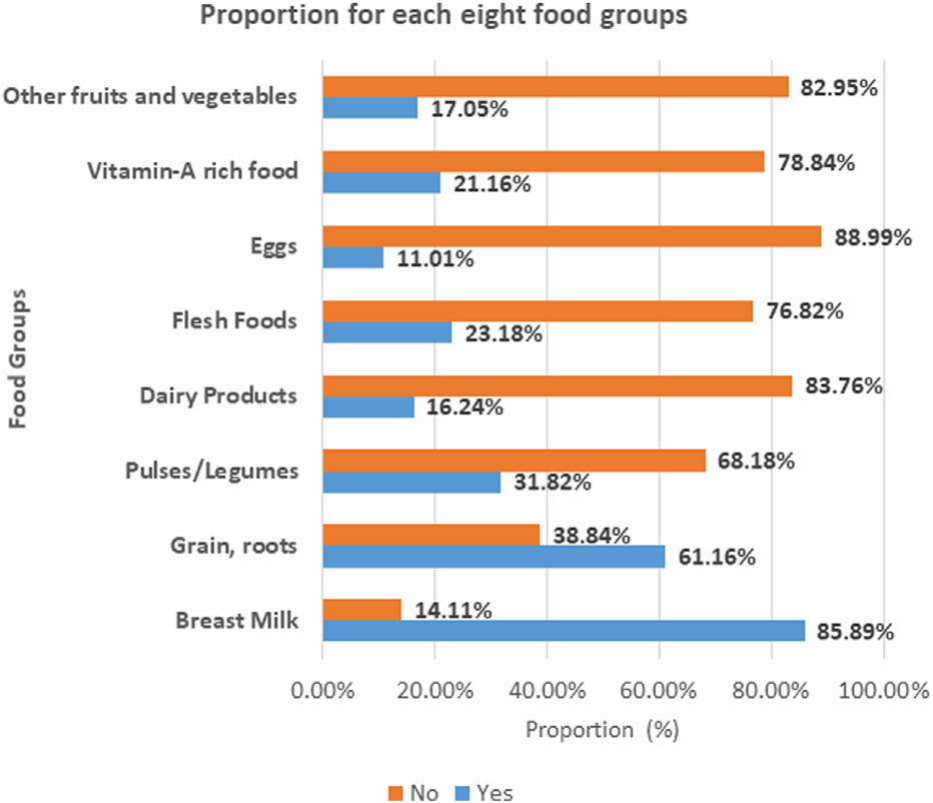
The proportion of each of the eight food groups in East Africa (2015–2020).

The following figure presents the proportion of dietary consumption for each of the eight food groups. The majority of children aged 6–59 months in East Africa (88.99%) and (83.76%) cannot eat eggs and dairy products, respectively. However, children aged 6–59 months in East Africa have better access to breast milk (85.89%) and cereals and roots (61.16%) (Figure 1).

### Minimum Dietary Diversity Proportion

**FIGURE 2:**
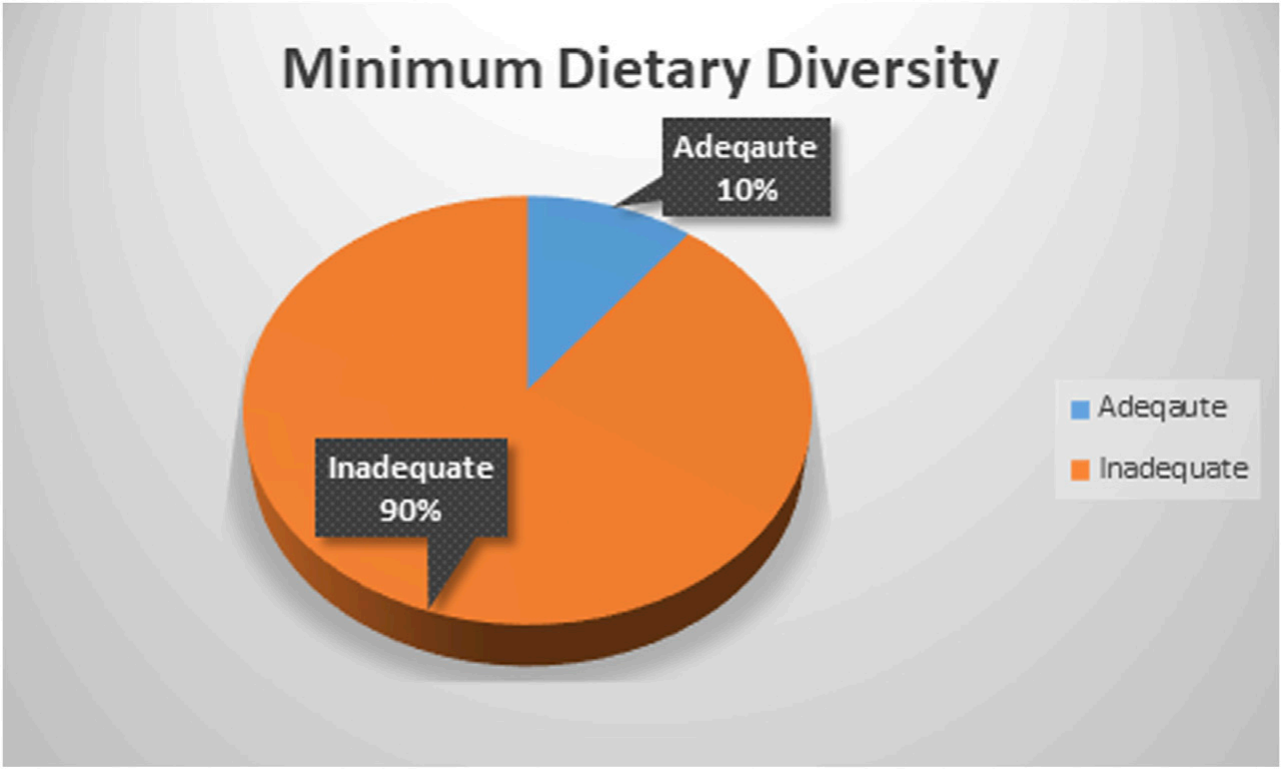
Magnitude of adequate minimum dietary diversity in East Africa (2015–2020).

The magnitude of adequate minimum dietary diversity in East Africa was found to be 10.47% with 95% CI (10.12–10.84) ranging from 6.81% with 95% CI (6.15–7.52) in Ethiopia to 16.22% with 95% CI (14.63–17.94) in Rwanda (Figure 2).

### Bi-Variable Model

Most of the children who had a mother aged 25–34 years had adequate MDD. The highest adequate MDD was reported for children whose mothers had a primary education and, educational attainment was significant in determining the MDD. In addition, the highest adequate MDD was reported among children whose families were rich and the wealth index was significant in determining the MDD (Table 3).

**TABLE 3:** Minimum Dietary Diversity of Children aged 6-59 months in East Africa countries from 2015 to 2020 and Bi-Variable Multilevel Mixed-Effect Logistic Regression in COR (*n* = 27,655).

Variables	Minimum dietary diversity		COR (95% CI)
	Not adequate	Adequate	
Age of Child (Months)			
6–23	14,593	1,985	1.80 (1.63–1.99)**
24–59	9,766	793	Ref.
Mothers Age (Years)			
15–24	7,396	9,820	Ref.
25–34	11,778	1,511	1.13 (1.01–1.27)*
35–49	5,196	521	0.82 (0.71–0.94)*
Mothers Education			
No Education	7,053	378	Ref.
Primary	12,294	1,317	1.97 (1.69–2.29)**
Secondary	4,548	918	4.14 (3.47–4.94)**
Higher	475	239	10.68 (8.05–14.16)**
Mothers occupation			
No Occupation	8,100	783	Ref.
Had Occupation	16,270	2,069	1.38 (1.23–1.56)**
Sex of HH Head			
Female	4,847	476	Ref.
Male	19,523	2,376	1.44 (1.26–1.63)**
Wealth Index			
Poor	12,318	792	Ref.
Middle	4,916	443	1.44 (1.24–1.67)**
Rich	7,136	1,617	3.75 (3.28–4.29)**
Media Exposure			
No	10,744	670	Ref.
Yes	13,626	2,182	2.50 (2.22–2.82)**
Partner Education (*n* = 24,209)			
No Education	5,122	272	Ref.
Primary	10,878	1,164	1.93 (1.62–2.29)**
Secondary	4,791	821	3.41 (2.80–4.15)**
Higher	829	332	8.94 (6.86–11.64)**
Number of HH Members			
≤5	10,688	1,447	Ref.
6 and Above	13,682	1,405	0.77 (0.70–0.85)**
Number of under 5 children			
≤2	18,344	2,397	Ref.
3 and Above	6,026	455	0.55 (0.49–0.63)**
Age at 1st birth			
<20	14,640	1,395	Ref.
20–30	9,518	1,409	1.55 (1.39–1.71)**
31–49	212	48	1.91 (1.22–2.98)**
Community Level Education			
Lower	6,019	360	Ref.
Higher	18,351	2,492	2.21 (1.79–2.70)**
Residence			
Rural	20,295	1,834	Ref.
Urban	4,075	1,018	3.55 (2.96–4.26)**
Country			
Burundi	3,281	311	1.48 (1.04–2.10) *
Ethiopia	4,813	352	Ref.
Malawi	2,048	340	3.14 (2.23–4.41)**
Rwanda	1,588	307	4.14 (2.89–5.92)**
Tanzania	4,407	531	2.70 (1.94–3.76)**
Uganda	2,056	345	2.79 (1.97–3.3.95)**
Zambia	3,949	421	1.58 (1.12–2.23)**
Zimbabwe	2,228	245	1.77 (1.21–2.59)**
Number of ANC Visit			
No Visit	11,436	931	Ref.
1–3 Visit	5,550	717	1.66 (1.47–1.88)**
4 and above visit	7,384	1,204	1.98 (1.77–2.21)**
Child Twin			
No	23,782	2,803	Ref.
Yes	588	49	0.51 (0.35–0.73)**
Birth Order			
First Order	5,563	787	Ref.
Two to Fourth order	11,670	1,433	0.83 (0.74–0.92)**
Fifth and above order	7,137	632	0.64 (0.56–0.74)**
Place of Delivery			
Home	8,174	486	Ref.
Health Institution	16,196	2,366	2.17 (1.88–2.50)**
Post-natal check with in 2 months (*n* = 16,189)			
No	9,142	1,164	Ref.
Yes	5,023	862	1.30 (1.15–1.46)**
Meal Frequency (*n* = 16,458)			
One Time	3,464	328	Ref.
Two Time	5,340	649	1.38 (1.17–1.62)**
Three Time	3,791	628	2.14 (1.80–2.54)**
Four and above	1,613	423	3.44 (2.84–4.17)**

The symbol “**” implies the statistical significance with p-value less than 0.001 and the symbol “*, **” implies the statistical significance with p-value less than 0.05.

### Determinants of Dietary Diversity

#### Random Effects

The results of the null model revealed that there was statistically significant variability in the odds of MDD with community variance (S.E.) of 3.47 (0.20). Likewise, the ICC in the null model showed that 51.38% of the total variance was due to differences between communities. The MOR was also 5.87 which is significant. This suggests that the likelihood of having adequate MDD was 5.87 times higher when respondents moved from low-risk to high-risk communities. This has shown a significant heterogeneity in MDD between different communities. In the full model (adjusted for individual and community factors), the community variance (community variance = 1.56; SE = 0.14) remained significant but decreased. Approximately 32.20% of the total variance of adequate MDD that can be attributed to contextual factors remained significant even after accounting for some contextual risk factors. The PCV in this model was 55.04%, indicating that 55.04% of the community variance observed in the null model was explained by the community as well as individual variables (Table 4).

**TABLE 4:** Multivariable multilevel mixed-effect logistic regression analysis of determinants of dietary diversity in East Africa countries from 2015 to 2020.

Variables	Models			
	Null model	Model-I	Model-II	Model-II
	AOR (95% CI)	AOR (95% CI)	AOR (95% CI)	AOR (95% CI)
Age of Child (Months)				
6–23	—	1.33 (0.18–9.96)	—	1.21 (0.16–9.11)
24–59	—	Ref.	—	Ref.
Child Twin				
No	—	Ref.	—	Ref.
Yes	—	0.59 (0.33–1.05)	—	0.55 (0.31–0.98)*
Mothers Age (Years)				
15–24	—	Ref.	—	Ref.
25–34	—	1.01 (0.84–1.21)	—	1.02 (0.84–1.22)
35–49	—	0.70 (0.54–0.92)*	—	0.70 (0.53–0.91)*
Mothers Education				
No Education	—	Ref.	—	Ref.
Primary	—	1.47 (1.20–1.80)**	—	1.41 (1.12–1.77)**
Secondary	—	1.90 (1.48–2.46)**	—	1.98 (1.50–2.63)**
Higher		3.03 (2.02–4.53)**	—	3.04 (1.99–4.64)**
Mothers occupation				
No Occupation	—	Ref.	—	Ref.
Had Occupation	—	1.41 (1.22–1.62)**	—	1.29 (1.11–1.49)**
Birth Order				
First Order	—	Ref.	—	Ref.
Two to Fourth order	—	0.99 (0.83–1.20)	—	1.01 (0.84–1.22)
Fifth and above order	—	1.24 (0.93–1.64)	—	1.28 (0.96–1.70)
Sex of HH Head				
Female	—	Ref.	—	Ref.
Male	—	1.42 (1.18–1.70)**	—	1.41 (1.17–1.69)**
Wealth Index				
Poor	—	Ref.	—	Ref.
Middle	—	1.29 (1.07–1.54)*	—	1.27 (1.05–1.52)*
Rich	—	2.07 (1.74–2.46)**	—	1.85 (1.53–2.24)**
Media Exposure				
No	—	Ref.	—	Ref.
Yes	—	1.53 (1.32–1.79)**	—	1.44 (1.23–1.68)**
Partner Education				
No Education	—	Ref.	—	Ref.
Primary	—	1.29 (1.04–1.60)*	—	1.25 (1.01–1.55)*
Secondary	—	1.38 (1.07–1.77)*	—	1.50 (1.16–1.93)**
Higher	—	2.00 (1.42–2.83)*	—	2.03 (1.43–2.89)**
Number of HH Members				
≤5	—	Ref.	—	Ref.
6 and Above	—	0.92 (0.79–1.07)	—	0.81 (0.85–1.10)
Number of under 5 children				
≤2	—	Ref.	—	Ref.
3 and Above	—	0.86 (0.71–01.06)	—	0.88 (0.72–1.08)
Age at 1st birth				
<20	—	Ref.	—	Ref.
20–30	—	1.38 (1.19–1.59)**	—	1.32 (1.14–1.52)**
31–49	—	1.41 (0.80–2.47)	—	1.28 (0.73–2.25)
Number of ANC Visit				
No Visit	—	Ref.	—	Ref.
1–3 Visit	—	1.03 (0.75–1.40)	—	0.92 (0.66–1.26)
4 and above visit	—	0.99 (0.0.73–1.36)	—	0.95 (0.69–1.30)
Place of Delivery				
Home	—	Ref.	—	Ref.
Health Institution	—	1.36 (1.13–1.65)**	—	1.26 (1.04–1.54)*
Post-natal check with in 2 months				
No	—	Ref.	—	Ref.
Yes	—	1.09 (0.95–1.26)	—	1.24 (1.07–1.45)**
Meal Frequency				
One Time	—	Ref.	—	Ref.
Two Time	—	1.38 (1.15–1.65)**	—	1.37 (1.14–1.64)**
Three Time	—	2.07 (1.72–2.50)**	—	2.09 (1.73–2.52)**
Four and above	—	2.83 (2.29–3.50)**	—	2.96 (2.39–3.66)**
Community Level Education				
Lower	—	—	Ref.	Ref.
Higher	—	—	1.60 (1.30–1.97)**	0.95 (0.72–1.25)
Residence				
Rural	—	—	Ref.	Ref.
Urban	—	—	3.62 (3.02–4.34)**	1.33 (1.08–1.63)**
Country				
Ethiopia	—	—	Ref.	Ref.
Burundi	—	—	1.47 (1.05–2.07)*	1.29 (0.91–1.83)
Malawi	—	—	2.92 (2.09–4.08)**	2.04 (1.44–2.89)**
Rwanda	—	—	3.52 (2.48–5.01)**	2.08 (1.44–3.00)**
Tanzania	—	—	2.17 (1.58–2.99)**	1.45 (1.05–2.01)*
Uganda	—	—	2.38 (1.70–3.35)**	1.63 (1.15–2.33)**
Zambia	—	—	1.13 (0.82–1.59)	0.77 (0.54–1.09)
Zimbabwe	—	—	1.23 (0.85–1.79)	0.65 (0.44–0.96)*
Random Effects				
Community Variance (SE)	3.47 (0.22)	1.63 (0.15)	2.74 (0.18)	1.56 (0.14)
ICC(%)	51.38%	33.17%	45.45%	32.20%
PCV(%)	Ref.	53.02%	21.04%	55.04%
MOR	5.87	3.36	4.82	3.27
Model Comparison				
AIC	16060.65	9313.72	15750.3	**9251.57**
BIC	16077.07	9532.01	15840.6	**9531.60**

The bold value indicates AIC and BIC implies the Model with the smallest AIC and BIC which suggests it is the best parsimonious model in this study.

The symbol “**” implies the statistical significance with p-value less than 0.001 and the symbol “*, **” implies the statistical significance with p-value less than 0.05.

#### Fixed Effects

The model with smaller AIC and BIC was considered as a parsimonious model, and the interpretation of the fixed effects was based on this model. Model-III was adjusted for both individual and community-level factors that have small AIC and BIC, compared to other models, and this model fits the data well. In the multivariable analysis, respondent’s twin child, mother’s age, mother’s education, mother’s occupation, wealth index, sex of HH head, media exposure, partner education, age at 1st birth, place of delivery, meal frequency, place of residence, and country of origin were significant determinants of MDD in East Africa at a 5% level of significance.

The odds of having adequate minimum dietary diversity were 30% less likely among children whose mothers were in the age group of 35–49, compared to those whose mothers were in the age group of 15–19 (AOR = 0.70; 95% CI; 0.53–0.91). Twin children were 45% less likely to have adequate minimum dietary diversity, compared to their counterparts (AOR = 0.55; 95% CI; 0.31–0.91). Children whose mothers had higher educational attainment were 3.04 times more likely to have adequate minimum dietary diversity, compared to those whose mothers had no formal education (AOR = 3.04; 95% CI; 1.99–4.64). Children who had a post-natal check within 2 months had 24% higher odds of reaching adequate minimum dietary diversity, compared to their counterparts (AOR = 1.24; 95% CI; 1.07–1.45). Urban children had 33% higher odds of having adequate minimum dietary diversity, compared to the rural (AOR = 1.33; 95% CI; 1.08–1.63). The odds of reaching adequate minimum dietary diversity were 2.08 (AOR = 2.08; 95% CI; 1.44–3.00) times higher among the children who live in Rwanda, compared to those who live in Ethiopia. However, the odds of having adequate minimum dietary diversity were 0.65 (AOR = 0.65; 95% CI: 0.44–0.96) times lower among children living in Zimbabwe, compared to those living in Ethiopia.

## Discussion

This study assessed the minimum dietary diversity among children aged 6–59 months in East African Countries. This study found that the magnitude of Adequate minimum dietary diversity (MDD) among children aged 6–59 months was 10.47% (95% CI: 10.12–10.84), and women’s age, mother’s education level, mother’s occupation, partner educational level, media exposure, wealth index, place of delivery, meal frequency, residence, and country in which the mothers live determines the adequate MDD.

The magnitude of adequate dietary diversity among children aged 6–59 months in East Africa was found to be 10.47 (95% CI; 10.12–10.84). This finding is lower than the study conducted in different (sub-Saharan African countries (25.1%), (43.9%) in South Africa, (23.25%) in Ethiopia) [14], Dabat district (17%) in Ethiopia [23], Gedeo Zone (29.9%) [33] in Southern Ethiopia, Sinan district (13%) in Ethiopia [34], Dejen district (13.6%) in Ethiopia [4], Rwanda (23%), and Burundi (16%) [35], and it is also lower than the study conducted in Bangladesh (41.9%) [36] and India (15.2%) [37]. However, this finding is higher than a study conducted using 2011 EDHS in Ethiopia (10.8%) [38], EDHS 2016 (24%) in Ethiopia [39], Gorche district (10.6%) [40], and Burkina Faso (5.6%) [13].

The observed discrepancy in Minimum Dietary Diversity (MDD) rates may be attributed to various factors, including socioeconomic differences [41], variations in child feeding habits, differences in study settings, and variations in the age and size of study samples.

Socioeconomic differences play a significant role in shaping child feeding habits and dietary patterns. Communities with different economic conditions may have distinct food availability, access, and cultural preferences, which can influence the diversity of foods consumed by children [41].

Child feeding habits can also vary across societies based on their dominant agricultural practices or reliance on animal products. Some communities may have a tradition of predominantly plant-based diets, while others may heavily rely on animal-derived foods.

The differences in study settings, such as geographical location or cultural context, can further contribute to variations in child feeding practices. These variations may be influenced by regional traditions, dietary norms, and availability of food resources.

Furthermore, the age range and sample size of the study can impact the findings. Different age groups may have different dietary needs and behaviors, leading to variations in MDD rates. Additionally, the size of the study sample can affect the generalizability of the findings to the larger population.

The lower proportion of MDD observed in the present study aligns with the findings from the Afrobarometer SDG Scorecards report. The report indicates a decline in performance, by more than 3 percentage points, in achieving targets related to SDG1 (no poverty), SDG2 (zero hunger), and SDG3 (good health and wellbeing) in most East African countries between the 6th and 8th round surveys conducted from 2014/15 to 2019/21 [42].

Overall, these factors collectively contribute to the observed discrepancies in MDD rates, highlighting the complex interplay between socioeconomic factors, cultural practices, and regional trends in achieving sustainable development goals related to poverty, hunger, and health. Addressing these variations and challenges requires targeted interventions and policy efforts to improve child nutrition and wellbeing in diverse.

The odds of having adequate dietary diversity were 30% lower among children whose mothers were in the age group of 35–49, compared to those whose mothers were in the age group of 15–19, which is supported by a study done in Ethiopia [23] and Bangladesh [43]. As the age of the mother increased, the probability of children getting adequate minimum dietary diversity of food is decreased [40, 43]. This may be related to the increase in maternal age and increase in family size that can affect the economic status of households to provide a variety of nutrients for all family members as per recommendations. In addition, the effect of age on MDD was supported by the previous report which found that as children get older, their dietary diversity gradually increases [44]. Furthermore, this finding is crucial for policymakers to work effectively on meeting the SDGs target as adequate MDD is associated with a lower risk of stunted growth and wasting among children [13].

Children aged 6–59 months living in households within the rich wealth index were associated with significantly higher odds of adequate Dietary diversity compared to those living in households within the poor wealth index which is consistent with the study conducted in Ethiopia and other East African countries [38, 45, 46] and Bangladesh [36]. This may be related to higher economic status being associated with improved access to information, finance, and other resources which improve dietary diversity of the household. The lower wealth quintile was associated with unmet minimum dietary diversity and it is a limiting factor for the mother to provide an adequate variety of nutritious food to their children daily [47, 48]. Therefore, improvement in all wealth quintiles may have a significant effect on appropriate child-feeding practices to meet their nutritional requirements.

Children aged 0–59 months from mothers who delivered at health institutions were significantly associated with adequate dietary diversity. This is similar to studies conducted in Ethiopia [45]. This might be due to attending institutional delivery providing the opportunity of being counseled by health professionals on child care which influences child feeding.

This study indicated that children aged 6–59 months from mothers who lived in urban areas were significantly associated with adequate dietary diversity which converges with a study done in Indonesia [49]. This could be because individuals from urban areas are better educated, have better access to information or have better awareness, and have better access to health facilities which influences their child feeding practices [50]. Additionally, it is known that urban areas offer better opportunities to aid mothers’ understanding and access to a varied dietary diversity due to the cumulative effect of a series of more favorable conditions, including better socioeconomic and educational conditions, in turn leading to better caring practices for children [51]. Thus, children who lived in rural residences can have a higher probability of inadequate MDD.

In this study, exposure to mass media showed a significant association with adequate dietary diversity among children aged 6–59 months. This finding is consistent with a study conducted in Ethiopia [14, 23, 33, 34, 40, 45]. This might be due to nutritional-related health education and promotion delivered by mass media can improve the feeding practices of children as recommended. Another possible reason may be that having media access in the household can help mothers obtain information about varied food options for children. This indicates that mass media can be used as an effective source of information for promoting the feeding practice of infants and young children [14]. In addition, previous reports showed that Television media currently promotes and shows the practice of Infant and Young Child Feeding (IYCF) which includes breastfeeding, a healthy diet, and good nutrition for children [52]. Thus, this can increase food variations in children aged 6–23 months.

Mothers’ occupational and educational status had a significant association with achieving adequate minimum dietary diversity among children aged 6–59 months which parallels a study conducted in the east African region [24, 38, 46, 53]. This could be because a higher educational level allows mothers to have better knowledge about nutrition and food groups consumed by children [48]. Another justification might be that educated mothers have a better chance to be employed. This gives more opportunities to have a higher income for purchasing diverse foods for their children, to have more information and understand educational messages delivered through different media outlets that can have a positive impact on overall health and child feeding practice [10]. In addition, better education is also associated with women’s empowerment which will influence better care for their children.

Place of delivery was significantly associated with adequate child dietary diversity which was consistent with studies from Ethiopia [14, 34]. This might be because attending an institutional delivery may provide more opportunities to receive effective nutrition education and counseling provided during ANC visits and after delivery at health institutes that might contribute to being knowledgeable on dietary diversity [54]. Therefore, this may imply that encouraging the utilization of maternity services and integrating them with infant and young child feeding helps to improve a variety of diet supplementation and child feeding practices.

### Conclusion

The magnitude of adequate MDD among children aged 6–59 months in East Africa was found to be 10.47%. This finding is lower than the expected level. Therefore, more effort is needed in nutrition-specific interventions and strengthening the existing country-based strategies aimed to achieve the recommended minimum dietary diversity intake for all children aged between 6 and 59 months. Factors such as maternal education, maternal employment, exposure to media, wealth, post-natal checks, health institution delivery, and place of residence have a positive association with adequate MDD. However, having twin children and having more than five family members have a negative association with adequate MDD intake in East Africa.

Therefore, strengthening interventions focused on improving the economic status of households, the educational status of mothers, and diversified food consumption of children aged 6–59 months should be prioritized to improve the recommended feeding practice of children in east Africa. In addition, government support must be enhanced in all wealth quintiles to ensure that children receive a variety of foods. Furthermore, future country-based studies may be needed to examine the barriers to adequate MDD specifically in each country for better specific recommendations.

## Data Availability

Data for this study were sourced from Demographic and Health surveys (DHS) and available at: https://www.dhsprogram.com.

## Abbreviations

AIC, akaike information criteria; ANC, ante-natal care; AOR, adjusted odds ratio; BIC, Bayesian information criteria, CI, confidence interval; COR, crude odds ratio; DHS, demographic and health survey; ICC, intra-cluster correlation coefficient; MDD, minimum dietary diversity; MOR, median odds ratio; SDGs, sustainable development goals; SSA, Sub-Saharan Africa; PCV, proportional change in variance; WHO, world health organization.
